# Neural Correlates of Letter Reversal in Children and Adults

**DOI:** 10.1371/journal.pone.0098386

**Published:** 2014-05-23

**Authors:** Liwei King Blackburne, Marianna D. Eddy, Priya Kalra, Debbie Yee, Pawan Sinha, John D. E. Gabrieli

**Affiliations:** 1 Department of Brain and Cognitive Sciences, Massachusetts Institute of Technology, Cambridge, Massachusetts, United States of America; 2 McGovern Institute for Brain Research, Massachusetts Institute of Technology, Cambridge, Massachusetts, United States of America; 3 Harvard Graduate School of Education, Cambridge, Massachusetts, United States of America; The Ohio State University, Center for Cognitive and Brain Sciences, Center for Cognitive and Behavioral Brain Imaging, United States of America

## Abstract

Children often make letter reversal errors when first learning to read and write, even for letters whose reversed forms do not appear in normal print. However, the brain basis of such letter reversal in children learning to read is unknown. The present study compared the neuroanatomical correlates (via functional magnetic resonance imaging) and the electrophysiological correlates (via event-related potentials or ERPs) of this phenomenon in children, ages 5–12, relative to young adults. When viewing reversed letters relative to typically oriented letters, adults exhibited widespread occipital, parietal, and temporal lobe activations, including activation in the functionally localized visual word form area (VWFA) in left occipito-temporal cortex. Adults exhibited significantly greater activation than children in all of these regions; children only exhibited such activation in a limited frontal region. Similarly, on the P1 and N170 ERP components, adults exhibited significantly greater differences between typical and reversed letters than children, who failed to exhibit significant differences between typical and reversed letters. These findings indicate that adults distinguish typical and reversed letters in the early stages of specialized brain processing of print, but that children do not recognize this distinction during the early stages of processing. Specialized brain processes responsible for early stages of letter perception that distinguish between typical and reversed letters may develop slowly and remain immature even in older children who no longer produce letter reversals in their writing.

## Introduction

Parents and teachers often observe that young children reverse individual letters when learning to read and write. Such letter reversal occurs both for letters that are mirror images of one another, such as *b* and *d*, and for letters for which reversals do not exist, such as *k* or *r*. These latter reversals are especially striking because children are producing letters that they have never observed in school or in books. Here, we used functional magnetic resonance imaging (fMRI) and event related potentials (ERPs) to compare brain activity between children, ages 5–12, and young adults as they viewed typical and reversed letters in order to delineate the brain basis of such letter reversals in children.

Letter reversal in reading and writing is common in beginning readers. The phenomenon was once thought to be a hallmark of dyslexia, but evidence for a selective propensity for such reversals in dyslexia is mixed [1], [2]. Some studies have found that children with dyslexia display more letter reversal errors [2]–[5], but other studies have found either no or very little difference in such errors between normal-reading and dyslexic children [6], [7]. Regardless of the inconclusive findings regarding dyslexia, it is clear that letter reversals commonly occur in non-dyslexic beginning readers. For example, children between the ages of three and seven will often spontaneously write backwards if asked to write their name next to the right-hand margin of a sheet of paper, flipping both the order of letters as well as the orientation of the letters themselves [8]. As children become more skilled at reading, reversal errors decrease.

One hypothesis for the frequency of letter reversal in children is that learning to read reflects a specialized adaptation of more general object recognition processes that are insensitive to right-left orientation [9], [10]. For purposes of object recognition, generalization across different appearances or perspectives may be helpful (e.g., a dog is a dog regardless of whether the dog is facing to the left or the right). For letters in an alphabet, however, specific right-left orientation is often definitional of the letter (e.g., a *b* vs. a *d*, or a *p* vs. a *q*). Thus, if learning to read letters reflects a specialized skill that is adapted from more general object recognition processes, then reading experience is needed to overcome the initial propensity to disregard right-left orientation. The idea that reading experience is needed to overcome orientation insensitivity is supported by the slow development of orientation specificity in children. Furthermore, learned orientation sensitivity for reading may promote orientation sensitivity for objects: Adults who were literate in a language where mirror orientation mattered for letter identity were more likely to reject mirror image *objects* in a matching task than adults who were literate in a language where mirror orientation does not matter for letter identity [11], [12].

Neuroimaging evidence also suggests that writing systems may be a special case for mirror reversal. Repetition priming studies in adults of the visual word form area (VWFA), an area of the left fusiform gyrus shown to be important for reading [13]–[15], have found that the region generalizes between mirror images of objects, but not of words [10] or letters [16]. In addition, studies using event-related potentials (ERPs) to examine the time-course of letter perception have found that letter reversals lead to an increased ERP amplitude for processing reversed relative to typically oriented letters in adult readers [17], [18]. These studies focused on later ERP components that likely reflect mental rotation, but orientation information ought to be important also in early stages of the visual processing of letters and words. In support of this idea, one study found that orientation of letters influenced the amplitudes of early ERP components, including the P1 (which is associated with low-level visual features) and the N170 (which is associated with categorization/classification processes) [19]. Both the P1 and N170 have posterior distributions, likely reflecting generators in primary visual cortex and ventral temporal cortices [20].

To the best of our knowledge, however, there is no evidence as to whether letter orientation is processed similarly or dissimilarly in the brains of children and adults. Here, we compared children, ages 5–12, and young adults viewing typical and reversed letters as we recorded fMRI and ERPs to examine the location and time course (respectively) of differential responses to typical and reversed letters. We performed whole-brain fMRI analyses on each participant. In addition, we examined fMRI responses in the VWFA as an *a priori* region of interest (ROI) identified in each participant in an independent localizer task. We chose to examine the VWFA in particular because it has been shown in numerous studies to be involved in visual word processing. Meta-analyses have found that region activates reliably to visually presented words [21], and that activation is consistent across tasks and different types of writing systems (both phonetic and logographic) [22], [23]. The region displays several characteristics useful for visual word processing, including location invariance, the ability to generalize across letter case [13], [24] but see [25], and a preference for known scripts over unknown scripts [26].

In the ERP portion of the study, we expected that the P1 and N170 responses should show sensitivity to orientation information about letters, because the P1 is sensitive to low level visual features important for identifying stimuli, and the N170 is sensitive for stimulus categorization and has been show to change with the acquisition of reading skills [27]. Importantly, these components should show differences between children and adults on the basis of experience with reading.

## Materials and Methods

### fMRI Experiment

#### Participants

Participants were right-handed English speaking children and adults with no history of reading difficulty, who were recruited from the university and surrounding community. Participants were required to have been exposed to English from birth, and not to have been exposed to any other language before the age of two. Written informed consent for participation in the study, approved by the MIT Institutional Review Board, was obtained from all adult participants and from the legal guardians of child participants. Verbal assent was obtained from all children, and additional written assent for children who could read and write. Adults were compensated for their participation and children received gift cards to a bookstore for participating.

Participants were chosen from among a larger group of participants (N = 76, 37 adults). Inclusion/exclusion criteria were applied to ensure that each participant understood and performed the scanner tasks and that all participants were typically developing for reading and reading-related skills. Children and adults met the following criteria: 1) For scanner behavioral performance, had an overall accuracy >70% and detected over 70% of target stimuli in both a localizer and the letter reversal task, 2) Scored above a 90 standard score on the Woodcock Reading Mastery Tests (WRMT)[28] and Test of Word Reading Efficiency (TOWRE)[29], above a standard score of 6 or greater on the Elision, Memory for Digits, Nonword Repetition, and Blending Words subtests of the Comprehensive Test of Phonological Processing (CTOPP) [30], and above a standard score of 85 on the Kaufman Brief Intelligence Test (KBIT) [31]. All children meeting these criteria were included in the study, and 15 adults were chosen so that the two groups were matched for KBIT score. The final group consisted of 15 children (9 male, mean age 9.5, age range 5–12) and 15 adults (N = 15, 7 male, mean age 22.3, age range 18–26). Behavioral scores are summarized in Table 1.

**Table 1:** Behavioral Scores for Participants in fMRI Experiment.

	Adults		Children		
Test	*M*	*SD*	*M*	*SD*	p value
**KBIT Nonverbal**	114.47	8.70	120.27	13.54	ns
**WRMT Word ID**	107	6.07	122.33	17.04	<.05
**WRMT Word Attack**	104	8.90	120.40	13.16	<.001
**TOWRE SWE**	106.27	9.00	117.87	11.38	<.05
**TOWRE PDE**	105.53	8.46	119.67	8.23	<.001
**CTOPP Elision**	10.93	1.10	13.27	2.19	<.001
**CTOPP Memory for Digits**	11.07	3.80	11.47	5.04	ns
**CTOPP Nonword Repetition**	9.13	1.64	9.80	2.11	ns
**CTOPP Blending Words**	12.47	1.13	11.73	2.25	ns

This table is reporting standard scores, except of the CTOPP where we report scaled scores, therefore they do not have the typical mean of 100 like standard scores, instead they have mean of 10 and a standard deviation of 3. KBIT  =  Kaufman Brief Intelligence Test; WRMT  =  Woodcock Reading Mastery Tests, CTOPP  =  Comprehensive Test of Phonological Processing; SWE  =  Sight Word Extraction; PDE  =  Phonemic Decoding Efficiency; ns  =  not significant. P values indicate significance level of t-test between the two groups on the measure.

#### Stimuli

**Figure 1:**
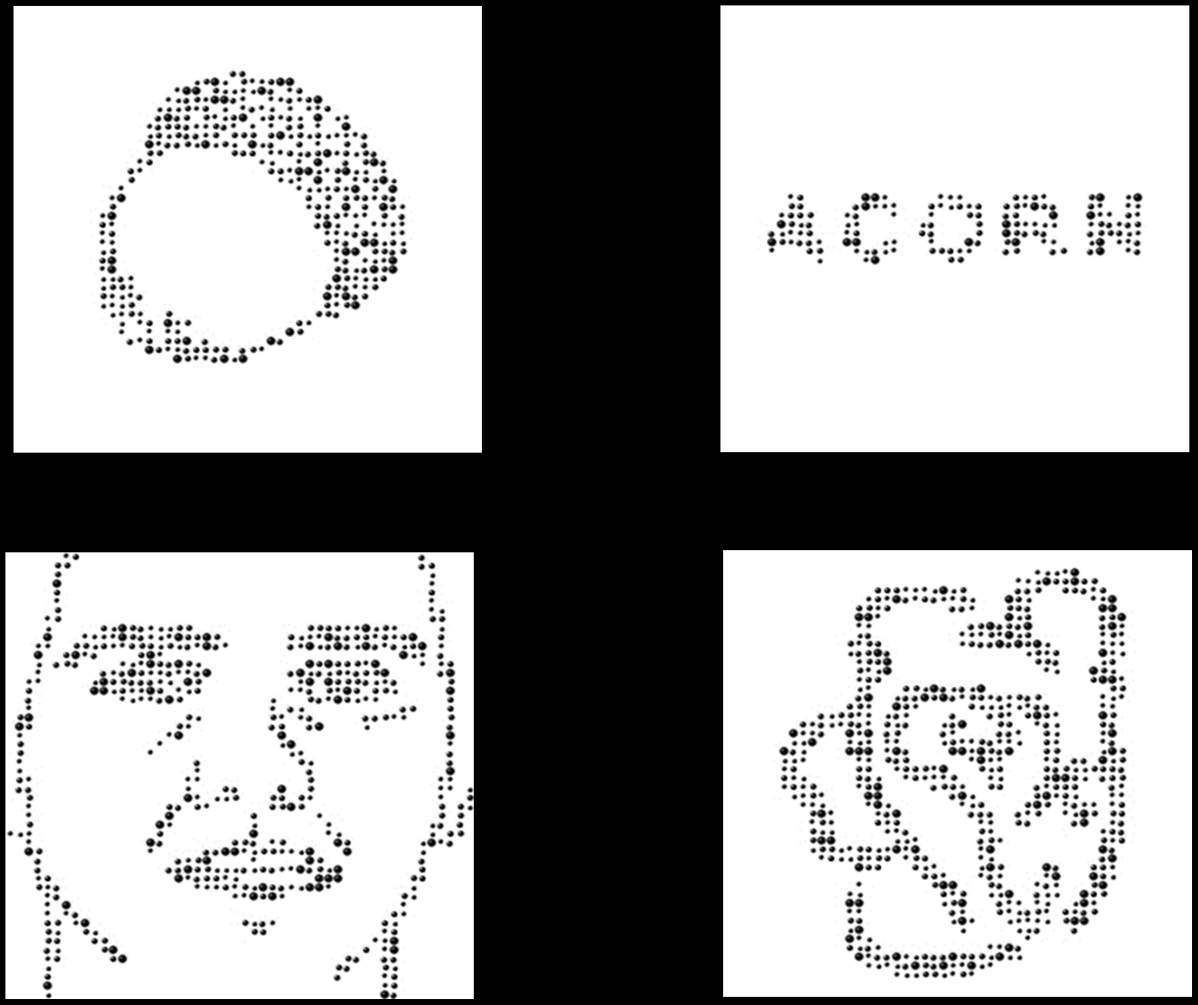
Stimuli for the Visual Word Form Area (VWFA) Localizer. Localizer stimuli consisted of four categories: objects, faces, words, and squiggles. Images were redrawn as dots to control for contour structure and spatial frequency.

VWFA Localizer: Stimuli consisted of words, drawings of faces, drawings of objects, and meaningless scribbles (196 each). To control for low-level visual characteristics (contour structure and spatial frequency), stimuli were constructed with a computer program that reconstructed the images as dot patterns (Figure 1). Words were nouns ranging from 3 to 8 letters long (average  = 4.6). Average Hyperspace Analogue to Language (HAL) frequency according to the English lexicon project was 27670 (SD  = 124497). Statistics for two words, ‘yoyo’, and ‘bagel’ were not available and thus were not included in the average. All stimuli were divided into two matched lists so that the words in one list were the names of the line drawings presented in the other list, and vice versa. Each participant viewed one list during the fMRI session and the other during the EEG session (EEG results for the localizer are not reported here). List assignment was counterbalanced between participants. Black and green versions of all stimuli were created for the task (described below). Stimuli were presented in a box that subtended about 4 degrees visual angle.

Letter Reversal Experiment: Stimuli for the letter reversal experiment consisted of lowercase letters, reversed letters, and pictures of chairs (16 each). The letters used were ‘a’, ‘c’, ‘e’, ‘f’, ‘g’, ‘h’, ‘j’, ‘k’, ‘m’, ‘n’, ‘r’, ‘s’, ‘t’, ‘u’, ‘y’, and ‘z’. Black and green versions of all stimuli were created. Stimuli were presented in a box that subtended approximately 4 degrees visual angle. Results from the two letter conditions are reported.

#### Procedure

VWFA Localizer: In each trial, participants were presented with a stimulus for 200 ms, followed by 800 ms of a blank screen. Stimuli were presented in black and white in a block design fashion, with each block consisting of 14 trials (14 s blocks). Participants were instructed to press the response button anytime a stimulus was green, which occurred one or two times per block. Between each block, a cartoon alien flashed on screen for 2 seconds. Because this paradigm was also performed with children, participants were told that the experiments were an attempt to teach the alien about color. Participants were scanned in this experiment for 2 runs of 4 minutes and 26 seconds each. In the two runs combined, there were 7 blocks of each condition, plus 6 fixation blocks.

**Figure 2:**
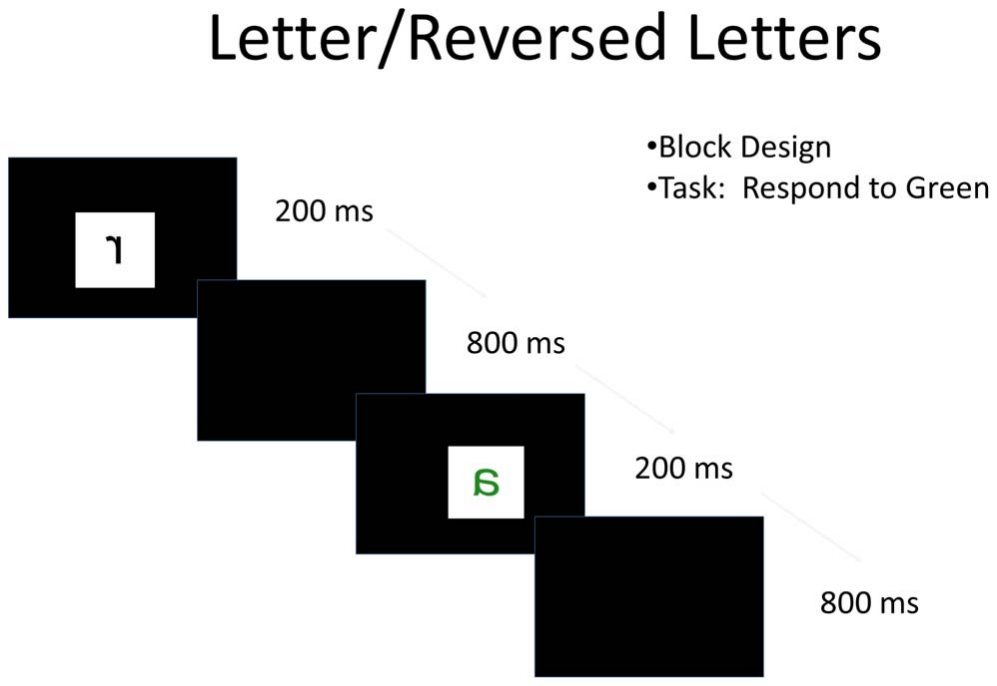
Letter reversal task. Participants were presented with a stimulus (letter, reversed letter, or chair) for 200 ms followed by 800 ms of a blank screen. Stimuli were presented in a block design in the fMRI portion, and an event related design in the ERP portion of the experiment.

Letter Reversal Task: As in the localizer task, stimuli in the letter-reversal task were also presented for 200 ms, followed by 800 ms of a blank screen (Figure 2). Stimuli were presented in blocks, each consisting of 16 trials, and like the localizer, there were one or two green stimuli per block. Participants were instructed to press the response button to any green stimulus. As in the localizer, an alien also flashed on the screen for 2 seconds between each block to keep the children engaged in the task. Participants were scanned for two runs of 4 minutes and 12 seconds each. In the two runs combined, there were 7 blocks of each condition, and 7 blocks of fixation (16 seconds long).

#### fMRI Acquisition and Analysis

fMRI scanning took place at the Athinoula A. Martinos Imaging Center at McGovern Institute for Brain Research at MIT. Imaging was performed using a Siemens 3T MAGNETOM Trio, A Tim System (Siemens Medical Solutions, Erlangen, Germany), and a commercial Siemens 32 channel head coil. High-resolution structural whole-brain images were acquired using a T1-weighted anatomical scan with motion correction (176 slices per slab; 1 mm isotropic voxel size; TR = 2530 ms; TE = 1.64 ms) [32].

Functional data were collected using a gradient echo T2*-weighted EPI sequence sensitive to the BOLD contrast (2 mm isotropic voxel size; TR  = 2; TE  = 30 ms; slices). Slices were placed at an oblique orientation parallel to the AC-PC line. We made sure that the lowest part of the occipital lobe and the bottom part of the temporal lobe in the left hemisphere (including the temporal pole) were covered. The uppermost part of the cortex in the frontal and parietal lobes were covered as well. Slices covered the entire cortex with the exceptions of the dorsal portion of the motor cortex in some participants and usually parts of the cerebellum.

The analysis was performed with SPM8, FreeSurfer, Artifact Rejection Toolbox (ART), and Advanced Normalization Tools (ANTS), using Nipype and bash scripts for workflow design and execution. Functional images were realigned to the mean image and smoothed with a 4 mm FWHM Gaussian kernel. The functional image co-registration to the 3D anatomical was performed in Freesurfer using a surface based registration algorithm. Structural and functional images were normalized to the Montreal Neurological Institute (MNI) space using ANTS [33]. Data were high-pass filtered with 128/s cutoff. In the first level analysis, each condition was convolved with a canonical HRF. A one-lag autoregression (AR(1)) model was used to correct for serial (i.e., temporal) autocorrelations. The ART toolbox was used to detect motion outliers. Timepoints whose position deviated from the previous by more than 1 mm, or whose average signal intensity deviated from the series average by more than 3 standard deviations, were added to the model as nuisance regressors. Realignment parameters were also added as nuisance regressors.

Whole brain random-effects analyses were performed by entering the SPM contrast images aligned to the subject specific ANTS normalized brain from the first level analysis into a second-level analysis of covariance, with stimulus correlated motion and number of artifacts as covariates. The ANTS normalization resampled the functional images to a voxel size of 1 mm^3^. Analyses were performed at a voxel-wise threshold of p<.01, with FDR cluster correction of p<.05 to control for multiple comparisons.

The words > object contrast at a p<.001 uncorrected threshold from the localizer paradigm was used to define a VWFA ROI in each individual's normalized functional scan. The closest cluster to the peak of the visual word form area from literature at −42 −57 −15 (Tal, converted to MNI -42 -58-21) [14] was selected. One child and two adults were excluded from this analysis because they did not have a cluster of greater than 5 voxels with a peak within 25 mm of those coordinates.

### EEG Experiment

Only the procedure and results of the letter reversal task are reported in this paper.

#### Participants

Of the 30 participants included in the fMRI experiment, 12 of the adults (4 males, mean age 22.3, age range: 18–26) and 10 of the children also participated in the EEG portion of the letter-reversal experiment. An additional two children who did not complete the fMRI experiment or were excluded from the fMRI analysis due to excessive motion were included in the EEG experiment for a total of 12 children (9 males, mean age of 9.4, age range: 7–12). The behavioral performance rates were slightly lower for the children in the EEG experiment due to pressing the wrong button on the response pad in some cases, however, all participants were video monitored during the experiment and were observed to be performing the task.

#### Stimuli

The stimuli for the EEG portion of the experiment were identical to the fMRI experiment.

#### Procedure

For the ERP version of the experiment, stimuli were presented for the same duration with the same inter-stimulus interval (ISI) as in the fMRI experiment; however, the stimuli were presented in event-related fashion by pseudorandomizing the order of presentation. The overall experiment time was the same as in the fMRI experiment because the fixation time was used as a time for blinking in the ERP experiment. The ERP task was identical to the MRI task with the same proportion of green items occurring and the stimuli broken up into two runs.

#### EEG Acquisition

A Biosemi ActiveTwo System (Biosemi B.V., Amsterdam, The Netherlands) using active Ag-AgCl electrodes mounted on an elastic cap (Electro-Cap, Inc.) was used to record from 61 scalp sites (10–20 system positioning), a vertical eye channel for detecting blinks, a horizontal eye channel to monitor for saccades, and two additional electrodes affixed the mastoid bone. The EEG was recorded with a low-pass hardware filter at 104 Hz and then digitized at 512 Hz with 24 bit of resolutions. Offline, all channels were filtered (bandpass 0.1–30 Hz) and referenced to a common average of scalp channels. Trials with blinks, eye movements, and muscle artifact were rejected prior to averaging.

#### ERP Analysis

For adults and children separately, ERP averages were formed by time-locking to the onset of the letters and reversed letters and averaging across these trials from 100 ms prior to target onset until 700 ms after (baseline −100 to 0). We examined three epochs where differences emerged in the ERP waveform across the two groups: the P1 (100–150 ms) and the N170 (150–225 ms). Mean amplitude measurements were taken from posterior electrode sites (P7, P5, P3, P8, P6, P4, PO3, PO4, PO7, PO8, O1, O2) for the P1 and measurements for the N170 were taken from P7/P8, PO3/PO4, and PO7/PO8 because the N170 tends to be maximal over occipito-temporal electrodes [34]. The ERP amplitude was normalized for both groups because children had much larger amplitude ERPs than adults, as is typical. This normalization was performed by taking the mean amplitude at one electrode site for one condition per participant (score) and subtracting the mean across all participants in each group from this score and dividing by the standard deviation across all participants in each group (score – mean/SD) (see [35]). This method eliminates main effects of group, but maintains all other main effects and interactions (analyses were performed with non-normalized data and the same pattern was found). The normalized mean amplitude from these electrode sites was entered into a repeated measures ANOVA with the within-subject factors of letter reversal (reversed or not reversed), electrode site (six levels for P1, three levels for N170), and hemisphere (left or right) and the between subject factor of group (adults, children). The Geisser and Greenhouse correction [36] was applied to all repeated measures having more than one degree of freedom and the corresponding p values are reported. When warranted by a condition x group interaction, follow-up analyses were conducted for each group separately on the non-normalized mean amplitudes with the same within-subject factors listed above. In addition a peak latency analysis was performed to examine differences in the timing between the groups and between the letter and reversed letter conditions. The peak latency was measured between 125 and 250 ms and only on negative going peaks that were the peak for at least +/− 5 consecutive points.

The non-normalized mean amplitude difference between reversed letters and letters from the ERP experiment at each of the left hemisphere electrodes (P7, PO3, PO7) was included in a correlational analysis with percent signal change difference between reversed letters and letters in the functionally defined VWFA for those participants who had both ERP data as well as a functionally defined VWFA in the fMRI portion of the experiment (N = 17, 10 adults, 7 children). We chose left hemisphere posterior-occipital electrodes since the VWFA is located in the left hemisphere.

## Results

### Behavioral Testing

Standardized reading and fluid intelligence measures for adults and children in the fMRI experiment are listed in Table 1. Adults scored higher than children on all measures analyzed as raw scores, but children had higher standardized (age-adjusted) scores than adults on several reading and reading-related measures. Importantly, all participants achieved scores on reading and reading-related measures that were in or above the normal range.

### fMRI Behavioral Performance

#### Localizer Task

Overall accuracy (measure includes correct rejections), percentage of probes detected, and reaction time for children and adults are reported in Table 2. Mixed model ANOVAs with 4 stimulus conditions as a within-subject factor and 2 age groups as a between subject factor were performed for each measure.

**Table 2:** Localizer Accuracy and Reaction Time.

**% Accuracy**	**Words**	**Faces**	**Objects**	**Scribbles**	**Overall**
Adults	99.5 (0.8)	99.6 (0.6)	99.7 (0.6)	99.8 (0.4)	99.7 (0.4)
Children	98.2 (2.7)	99.0 (1.8)	98.6 (2.1)	98.7 (2.3)	98.6 (2.1)
**% Probes Detected**	**Words**	**Faces**	**Objects**	**Scribbles**	**Overall**
Adults	97.0 (5.1)	98.5 (3.9)	97.8 (4.6)	98.5 (3.9)	98.3 (2.7)
Children	88.9 (17.3)	98.5 (3.9)	91.9 (12.2)	92.6 (10.0)	93.2 (7.1)
**Reaction Time (ms)**	**Words**	**Faces**	**Objects**	**Scribbles**	**Overall**
Adults	530 (61)	510 (45)	522 (57)	512 (54)	488 (133)
Children	705 (122)	665 (66)	676 (66)	666 (87)	671 (65)

Values listed are means, with standard deviation in parentheses.

Adults trended to be more accurate overall (99.7%) than children (98.6%) (F(1,28)  = 3.20, p = .09). The assumption of sphericity was violated for condition (chi-square  = 17.10, p = .004), and degrees of freedom were corrected using Greenhouse-Geisser estimates of sphericity (epsilon  = .77) and corrected p values are reported. There was trend toward a main effect of condition (F(3,84)  = 2.56, p = .08), and no condition by group interaction (F(3,84)  = 1.14, p = .33).

Adults detected a significantly higher percentage of probes (98.3%) than children did (93.2%) (F(1,28)  = 4.90, p = .04). The assumption of sphericity was violated for condition (chi-square  = 16.40, p = .006), and degrees of freedom were corrected using Greenhouse-Geisser estimates of sphericity (epsilon  = .74) and corrected p values are reported. There was a trend toward a main effect of condition, (F(3,84)  = 2.86, p = .06), and no condition by group interaction (F(3,84)  = 1.63, p = .20).

Adults responded faster to targets (488 ms) than did children (671 ms) (F(1,28)  = 48.12, p<.001). The assumption of sphericity was violated for condition (chi-square  = 18.50, p = .002), and degrees of freedom were corrected using Greenhouse-Geisser estimates of sphericity (epsilon  = .70) and corrected p values are reported. There was a trend toward a main effect of condition, (F(3, 84)  = 2.65, p = .08), and no condition by group interaction (F(3,84)  = 0.36, p = .70).

#### Letter Reversal Task

Adults had higher overall accuracy (99.9%) than children did (99.5%) (F(1,28)  = 6.3, p = .018). There was a trend toward higher accuracy in the typical letters condition, (F(1,28)  = 3.80, p = .06), and no condition by group interaction (F(1,28)  = 2.91, p = .10).

Letter reversal task results are reported in Table 3. Adults detected a significantly higher percentage of probes (98.9%) than children (94.4%) (F(1,28)  = 4.46, p = .044). There was no main effect of condition for probe accuracy (F(1,28)  = 0.84, p = .37), and no condition by group interaction (F(1,28)  = 0.30, p = .59).

**Table 3:** Letter Reversal In-Scanner and ERP Behavioral Data.

fMRI Behavioral Data			
**% Accuracy**	**Typical Letters**	**Reversed**	**Overall**
Adults	99.9 (0.3)	99.9 (0.2)	99.9 (0.2)
Children	98.9 (1.7)	99.8 (0.4)	99.5 (0.7)
**% Probes Detected**	**Typical Letters**	**Reversed**	**Overall**
Adults	98.5 (4)	99.3 (2.3)	98.9 (2.3)
Children	92.6 (12.4)	95.6 (10.1)	94.4 (8.1)
**Reaction Time (ms)**	**Typical Letters**	**Reversed**	**Overall**
Adults	473 (53)	487 (69)	480 (57)
Children	629 (71)	648 (74)	641 (63)

ERP Behavioral Data			
**% Accuracy**	**Typical Letters**	**Reversed**	**Overall**
Adults	92.9 (1.5)	94.7 (2.4)	93.8 (2.2)
Children	92.8 (1.2)	94.7 (1.8)	93.8 (1.8)
**% Probes Detected**	**Typical Letters**	**Reversed**	**Overall**
Adults	90.7 (10.4)	91.7 (13.5)	91.2 (11.8)
Children	68.5 (16.2)	74.1 (13.7)	72.9 (12.9)
**Reaction Time (ms)**	**Typical Letters**	**Reversed**	**Overall**
Adults	548 (43)	551 (64)	550 (52)
Children	603 (47)	585 (27)	593 (34)

Values listed are means, with standard deviation in parentheses.

Finally, adults responded faster to targets (480 ms) than children did (641 ms) (F(1,28)  = 52.74, p<.001). There was no main effect of condition for reaction time (F(1,28)  = 2.19, p = .15), and no condition by group interaction (F(1,28)  = 0.04, p = .85).

### Stimulus Correlated Motion and Number of Artifacts

Children averaged 19.27 (SD  = 15.88) rejected time points (7.24%) as defined in the methods section across both runs of the localizer task, while adults averaged 4.07 (SD  = 2.92) rejected time points (1.5%). Levene's test indicated that the two groups had unequal variances for number of rejected time points (F(28)  = 19.53; p<.001). An independent samples t-test (equal variances not assumed) showed that children had significantly more rejected time points than adults (t(14,94)  = 3.65, p = .002). Children (M = .095; SD  = .023) and adults (M = .11; SD  = .024) did not differ significantly (t(28)  = 1.49, p = .15) in the amount of stimulus correlated motion.

Children averaged 28.60 (SD  = 22.33) rejected timepoints (11.35%) as defined in the methods section across both runs of the letter reversal task, while adults averaged 5.67 (SD = 5.95) rejected timepoints (5.67%). Levene's test indicated that the two groups had unequal variances for number of rejected timepoints (F(28)  = 12.89; p = .001). An independent samples t-test (equal variances not assumed) showed that children had significantly more rejected timepoints than adults (t(15.98)  = 3.84; p = .001). Children (M = .087; SD  = .019) had significantly less stimulus correlated motion than adults (M = .12; SD  = .018) (t(28)  = 4.39, p<.001). Because children and adults differed in outliers and stimulus correlated motion, these parameters were added as covariates in the between groups whole-brain and ROI analyses.

### fMRI Whole-Brain Activations for Letter Reversal Task

**Figure 3:**
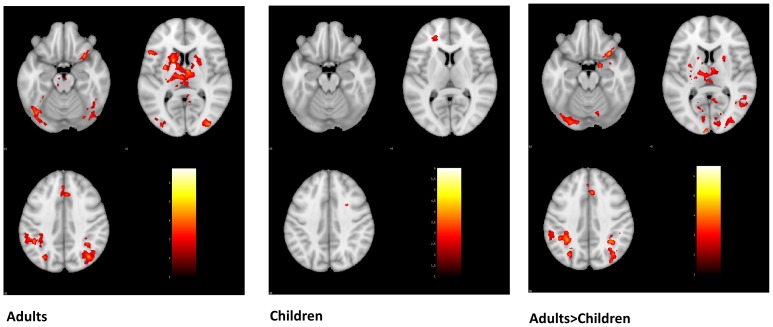
Reversed Letters > Typical Letters. Activation from the whole-brain analysis for the reversed letters > typical letters contrast. Direct comparison of children and adults showed that adults exhibited significantly greater activation for the reversed > typical letters contrast than children did in multiple regions, including the left ventral visual stream and bilateral parietal cortices.

We analyzed whole-brain results for the letter reversal task at a voxel-wise threshold of .01 with FDR correction of p<.05 (summarized in Table 4). Direct comparison of children and adults showed that adults exhibited significantly greater activation for the reversed > typical letters contrast than children did in multiple regions, including the left ventral visual stream and bilateral parietal cortices (Figure 3). Children did not exhibit greater activation than adults in the reversed > typical letters contrast.

**Table 4:** Whole Brain Activation.

**Normal Letter > Reversed: Adults**									
No activation									
**Normal Letter > Reversed: Children**									
**cluster FDR**	**size**		**peak T**		**x**		**y**	**z**	**Location**
0.034	1847		6.18		−31		−48	40	Parietal Lobe
			4.04		−30		−37	44	Parietal Lobe
			3.41		−34		−55	46	Inferior Parietal Lobule
0	4217		5.8		58		−41	10	Superior Temporal Gyrus
			5.14		49		−62	8	Middlemporal Gyrus
			3.81		51		−48	9	Superior Temporal Gyrus
0	6518		5.67		−5		101	6	Cuneus
			4.87		−10		−99	17	Cuneus
			4.02		−10		−96	−17	Lingual Gyrus
0	8995		4.73		7		−74	19	Precuneus
			4.65		8		−98	19	Cuneus
			4.6		6		−82	38	Precuneus
0.024	2082		4.22		−45		−35	23	Inferior Parietal Lobule
			3.67		−57		−46	16	Superior Temporal Gyrus
			3.35		−54		−38	27	Inferior Parietal Lobule
**Reversed> Normal Letters: Adults**									
**cluster FDR**	**size**		**peak T**		**x**		**y**	**z**	**Location**
0	8250		6.77		43		−73	−13	Middle Occipital Gyrus
			4.41		36		−86	9	Middle Occipital Gyrus
			4.27		49		−62	−11	Occipital Lobe
0	50298		6.57		−22		4	−7	Extra-Nuclear
			5.87		28		14	−13	Inferior Frontal Gyrus
			5.53		−26		−7	−3	Lentiform Nucleus
0.02	1960		6.03		40		8	32	Inferior Frontal Gyrus
			4.21		44		13	26	Frontal Lobe
			4.04		42		8	18	Frontal Lobe
0	11985		5.31		36		−50	47	Inferior Parietal Lobule
			5.28		37		−68	31	Angular Gyrus
			4.88		38		−77	31	Angular Gyrus
0	6253		5.11		−29		−48	43	Parietal Lobe
			4.84		−44		−45	40	Inferior Parietal Lobule
			3.95		−29		−69	39	Precuneus
0.001	3488		5.05		9		23	33	Cingulate Gyrus
			3.56		1		20	55	Superior Frontal Gyrus
			3.5		−1		35	38	Medial Frontal Gyrus
0.007	2510		4.39		−35		1	54	Middle Frontal Gyrus
			3.82		−23		15	59	Middle Frontal Gyrus
			3.43		−26		7	61	Middle Frontal Gyrus
0.01	2280		3.68		−4		−33	−30	Pons
			3.66		3		−23	−29	Pons
			3.59		8		−31	−29	undefined
**Reversed>Normal Letters: Children**									
**cluster FDR**	**size**		**peak T**		**x**		**y**	**z**	**Location**
0.039	1932		6.02		29		23	23	Frontal Lobe
			4.27		23		16	28	Frontal Lobe
			3.36		28		9	39	Frontal Lobe
0.039	2003		4.31		−26		40	−1	Middle Frontal Gyrus
			3.93		−22		45	5	Sub-Gyral
			3.79		−15		43	18	Medial Frontal Gyrus
**Reversed>Normal:Adults>Children**									
**cluster FDR**		**size**		**peak T**		**x**	**y**	**z**	**Location**
0	8252		6.52		−29		−48	42	Parietal Lobe
			4.5		−44		−45	40	Inferior Parietal Lobule
			4.36		−31		−37	45	Parietal Lobe
0	6139		6.33		38		−77	31	Angular Gyrus
			4.78		38		−68	31	Angular Gyrus
			4.48		12		−52	16	Posterior Cingulate
0	3852		5.83		36		−50	47	Inferior Parietal Lobue
			4.34		33		−50	38	Parietal Lobe
			3.72		50		−41	52	Inferior Parietal Lobule
0.007	2284		5.81		28		14	−14	Inferior Frontal Gyrus
			4.05		21		8	−11	Lentiform Nucleus
			3.96		38		24	−21	undefined
0.001	3414		4.86		9		23	34	Cingulate
			3.77		3		6	57	Superior Frontal Gyrus
			3.7		−1		27	56	Superior Frotnal Gyrus
0.015	1901		4.85		−2		−67	58	Precuneus
			4.12		13		−71	44	Precuenus
			3.34		7		−71	56	Superior Parietal Lobule
0	12146		4.64		34		−82	15	Middle Occipital Gyrus
			4.47		−5		−100	8	Cuneus
			4.08		−7		−94	−2	Cuneus
0.001	3488		4.55		−3		−23	4	Extra Nuclear
			3.98		6		−7	6	Thalamus
			3.87		−15		−18	−4	undefined
0.007	2262		4.42		−35		0	54	Middle Frontal Gyrus
			3.55		−22		12	59	Middle Frontal Gyrus
			3.46		−44		−3	55	Middle Frontal Gyrus
0.006	2378		4.39		26		18	−3	Claustrum
			4.23		37		28	2	Inferior Frontal Gyrus
			3.59		42		31	−5	Inferior Frontal Gyrus
0	5135		4.2		−22		4	−7	Extra Nuclear
			4.02		−24		−7	−1	Lentiform Nucleus
			3.75		−22		−31	0	Thalamus
0.046	1455		4.03		43		−3	45	Precentral Gyrus
			3.42		26		5	51	Sub-Gyral
			3.11		22		10	56	Superior Frontal Gyrus
0.005	2539		3.76		57		−56	23	Supramarginal Gyrus
			3.49		56		−53	15	Superior Temporal Gyrus
			3.4		48		−63	9	Middle Temporal Gyrus

**Figure 4:**
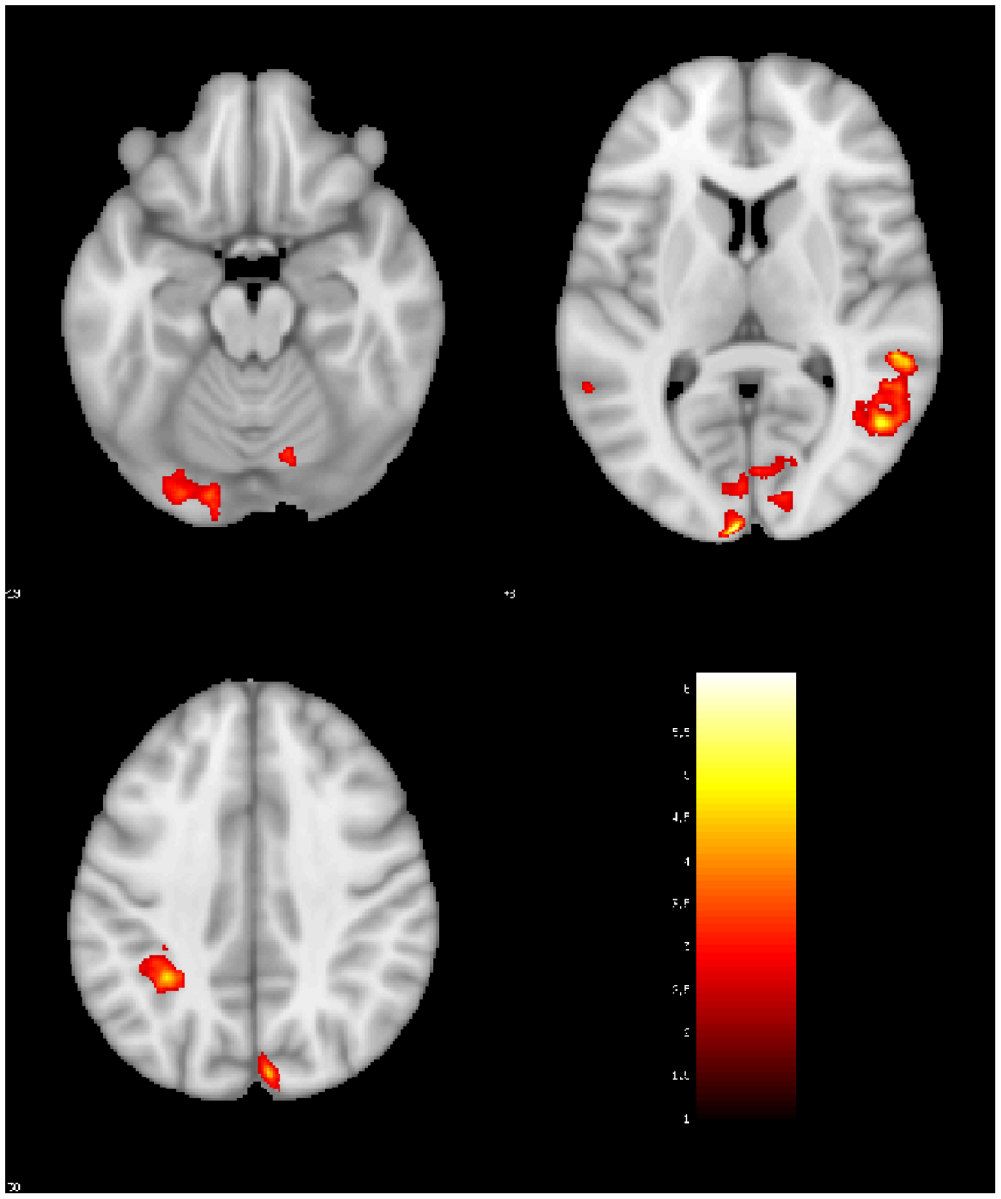
Typical Letters > Reversed Letters in Children. In children, there was greater activation for typical than reversed letters in left inferior parietal lobule, left superior temporal gyrus, and early visual regions. Adults had no activation for this contrast.

In adults, there was greater activation for reversed than typical letters in multiple regions, including the bilateral ventral visual stream, inferior frontal gyrus, angular gyrus, and inferior parietal lobule; no region exhibited greater activation for typical than reversed letters. In children, there was greater activation for typical than reversed letters in left inferior parietal lobule, left superior temporal gyrus, and early visual regions (Figure 4). Children showed greater activation for reversed letters than normal letters in the middle frontal gyrus.

### fMRI VWFA ROI Analysis

**Figure 5:**
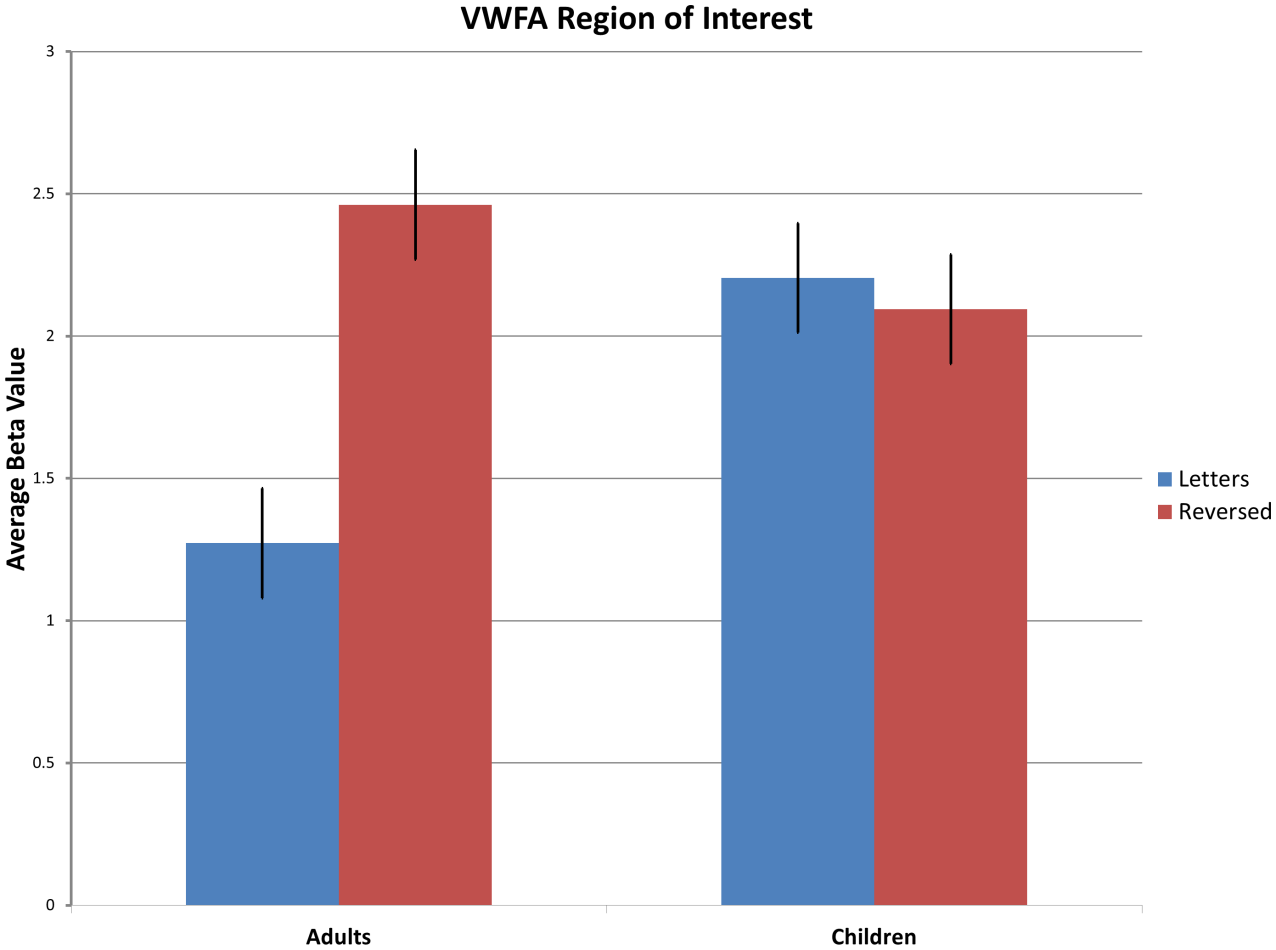
VWFA Region of Interest Analysis. Average beta values for independently defined VWFA region of interest. Adults had greater activation for reversed letters than letters, while children showed no difference. There was a significant interaction between group and letter type.

We extracted the average beta values in the *a priori* defined VWFA (Figure 5) in individual participants. We performed a repeated measures (adults/children group x typical/reversed letters) analysis of covariance (ANCOVA) with stimulus correlated motion and number of artifacts as covariates. There was no main effect of condition (F(1, 23)  = .05, p = .83) or group (F(1, 23)  = .757, p = .39). There was a significant interaction between group and letter type (F(1, 23)  = 7.77, p = .010). This interaction was further explored by comparing typical and reversed letters in adults and children separately. Adults showed significantly greater activation for reversed letters than typical letters (t(12)  = 2.59, p = .02). Children showed no activation difference between typical and reversed letters (t(13)  = .96, p = .35). Because the children covered a wide age range, we examined whether these effects correlated with age among the children. Age did not correlate with activation to letters (r(13)  = −.07, p = .81), reversed letters r(13)  = −.33, p = .24), or the difference between reversed and typical letters (reversed – letters) (r(13)  = −.41, p = .14).

### Matching for Excluded Timepoints

The above primary analyses included all 15 children and 15 adults. Children had more time points removed so as to minimize the effects of greater movement and other sources of artifact, and these parameters were further added to the group analyses. In order to make certain that these combined data-cleaning and statistical approaches to equating artifacts were effective, the same analyses were performed on a subgroup of children (N = 7, mean age  = 10.28, SD  = 1.65) and adults (N = 7, mean age  = 22.56, SD  = 2.55) who were chosen to be matched for the number of excluded time points (t(12)  = .99, p = .34) in the letter reversal task. In this sample, children had a mean of 12.71 (SD  = 6.02) excluded time points (5%) and adults had a mean of 9.29 (SD  = 6.92) excluded time points (3%). Children had a mean average stimulus correlated motion of .09 (SD  = .06) and adults had a mean stimulus correlated motion of .11 (SD  = .01). Children included in the subgroup trended toward a higher mean age (10.28) compared to children who were excluded (mean age  = 8.36) (t(12)  = 2.05, p = .06). There was no difference in ages between included (mean Age  = 22.56) and excluded (mean Age  = 21.89) adults (t(10)  = −.44, p = .67).

Results for the whole brain analysis were similar to those for the whole group, with fusiform activation for reversed > typical letters in adults, but not children, at a voxel-wise threshold of p <.01 and FDR correction of p<.05. The adults > children comparison for the same contrast and threshold also resulted in left fusiform activation. We also performed the same VWFA ROI analysis on this subset of participants. There was a main effect of condition (F(1,11)  = 5.82, p = . 03), no main effect of group (F(1,11)  = .22, p = .65), and a significant interaction between group and letter type (F(1,11)  = 10.34, p = .008). In adults, activation to reversed letters trended to be higher than typical letters (t(6)  = 1.93, p = .10), while children had no difference in activation (t(6)  = .787, p = .46, two-tailed).

### ERP Letter Reversal Task Performance

Letter reversal task results are reported in Table 3. Adults and children did not differ significantly on overall accuracy (F(1,22)  = 0.02, p = .88), however both groups were significantly more accurate in detecting mirror letter targets than typical letters (F(1,22)  = 9.04, p = .006) and there was no condition by group interaction (F(1,22)  = 0.04, p = .84).

Adults detected a significantly higher percentage of probes than children (F(1,22)  = 22.73, p<.001). There was no main effect of condition for probe accuracy (F(1,22)  = 0.78, p = .39), and no condition by group interaction (F(1,22)  = 0.4, p = .54).

Finally, adults responded faster to targets than children did (F(1,22)  = 6.17, p = .02). There was no main effect of condition for reaction time, (F(1,22)  = 1.02, p = .33), and no condition by group interaction (F(1,22)  = 2.23, p = .15).

### ERP Results

**Figure 6:**
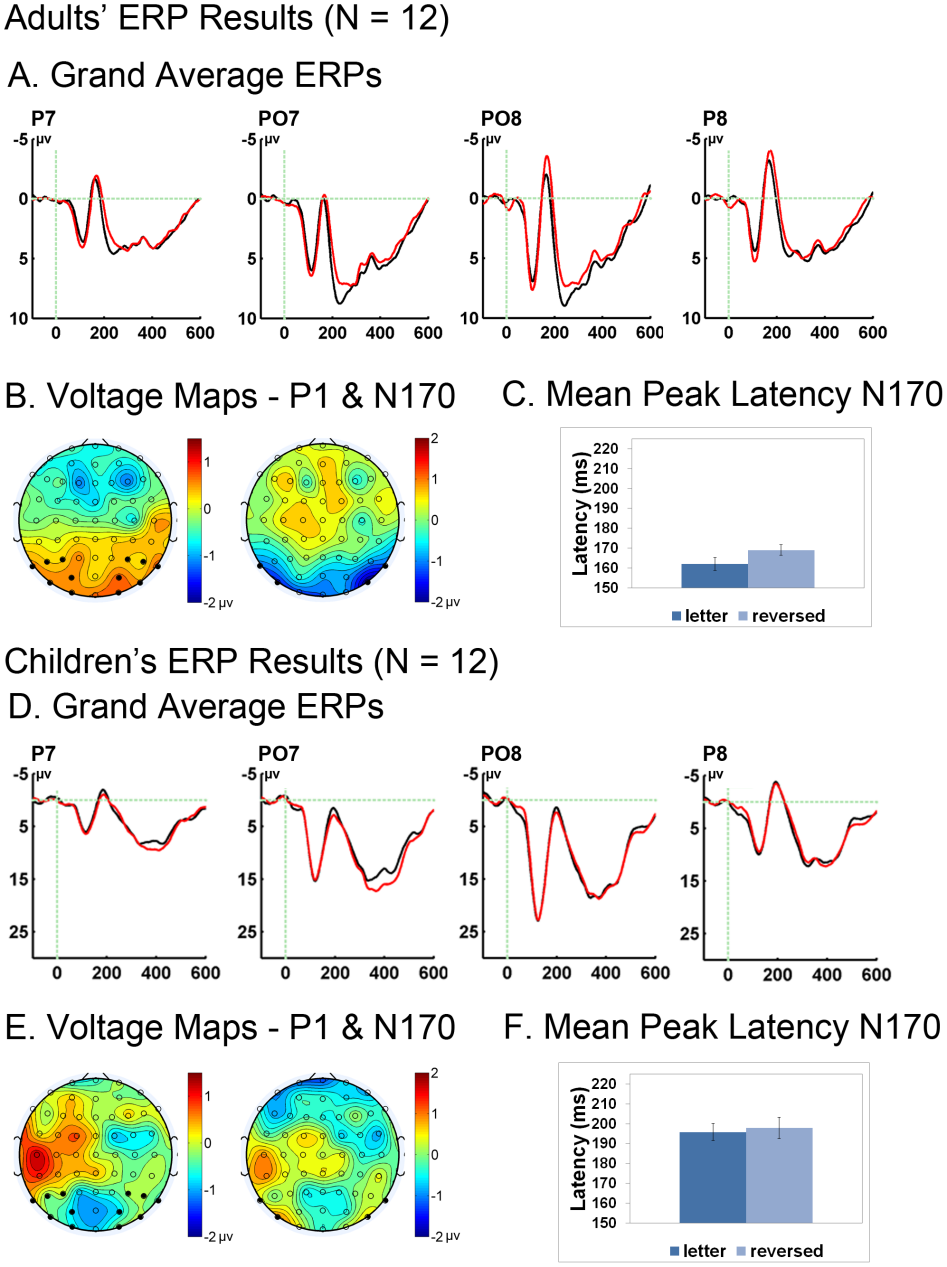
ERP Results. ERP Waveforms and Voltage Maps. Grand average waveforms for the adults (A) and for the Children (D) showing the P1 and N170 differences present in adults, but not children. The distribution of these effects is depicted in voltage maps (B and E) showing the difference between normally-oriented letters and mirror-reversed letters (reversed – normal). Black dots on the voltage maps indicate electrode sites included in the mean amplitude analysis. Note the scale difference between the P1 and N170 epoch. C shows the peak latency difference between reversed and normally-orientated letters in adults where the latency is increased for mirror reversed letters. In F, children show a delayed peak latency that does not differ between the two conditions.

ERP results are illustrated in Figure 6. Both adults and children exhibited a P1 and N170 to both typical letters and reversed letters. For the P1 component, adults had a significantly more positive going wave for reversed letters compared to typical letters than did children (group x condition interaction: F(1,22)  = 5.19, p = .03, *ηp^2^*  = .19). This interaction reflects the fact that adults showed a significantly more positive going amplitude for reversed compared to typical letters (F(1,22)  = 6.96, p = .02, *η ^2^*  = .39), whereas children showed no reliable difference between these conditions on this component (all F's <0.2, all p's >.60). The same pattern was also observed for the N170. The difference between typical and reversed letters varied between the adults and children in the directionality (group x condition interaction (F(1,22)  = 5.15, p = .03, *ηp^2^*  = .19). The N170 in adults was characterized by a more negative going wave for reversed letters than typical letters across the posterior electrodes (main effect of condition: F(1,11)  = 11.44, p = .006, *ηp^2^*  = .51), whereas in the children the reversed and typical letter conditions did not differ significantly in amplitude (F<0.41, p>.5).

The peak of the N170 occurred later in the children than in the adults (main effect of group: F(1,22)  = 36.25, p<.001, *ηp^2^*  = .62). This group difference interacted with whether or not the letters were reversed or normal (condition x group interaction: F(1,22)  = 6.31, p = .02, *ηp^2^*  = .22). Examining each group individually revealed that adults showed significant differences in the peak latency of reversed compared to normal letters (main effect of condition: F(1,11)  = 16.01, p = .002, *ηp^2^*  = .6), with reversed letters having a later peak latency than normally-oriented letter. Children did not show a difference in latency between the two letter conditions (all F's <1.1, all p's >.30).

### ERP and fMRI correlations

The mean amplitude difference on the N170 (computed by subtracting the amplitude of the response to typical letters from reversed letters) at electrode site P7 correlated significantly with the reversed letter > typical letter difference in the functionally defined VWFA (r(17)  = −.66, p = .004 (two tailed) (the correlation is negative because the N170 is a negative going effect) as well as with the mean amplitude difference at electrode site O1 (r(17)  = −.54, p = .03 (two-tailed)). Electrode site PO7 showed a marginally significant correlation with the VWFA activation (r(17)  = −.48, p = .05), whereas none of the other left hemisphere electrodes were significantly correlated. However, in each group separately there was not a correlation between ERP amplitude and VWFA activation, suggesting this correlation reflects the group difference observed on the N170.

## Discussion

We found major developmental differences in fMRI and ERP brain responses to typical versus reversed letters in children ages 5–12 and young adults. Adults exhibited widespread fMRI activation for reversed relative to typical letters, including left fusiform regions, associated with initial stages of reading. These activation differences were significantly greater than that exhibited by children; the children exhibited little difference in activation to reversed and typical letters. Adults also exhibited significant P1 and N170 ERP effects, with greater amplitude for reversed than typical letters, whereas children exhibited no reliable differences between the two letter types for these ERP components associated with early stages of visual processing. Thus, by every measure, adults showed significantly greater differences in brain responses to reversed relative to typical letters, whereas children showed little or no difference in brain responses to the two kinds of letters, or in fMRI an oppoosite difference of greater activation for typical than reversed letters.

The children and adults were well characterized. The two groups scored in the above average range on nonverbal IQ, reading, and reading-related language measures, and therefore represent unimpaired reading. The groups were similar on standardized (age-adjusted) nonverbal IQ. The adults had significantly better raw scores on all reading and reading-related measures, thus exhibiting the expected benefits of an average of about 13 more years of reading experience and other kinds of maturation. The two groups had similar age-standardized scores, and in the cases where the groups differed, the children exhibited a better score than the adults. Thus, it appears likely that the observed brain differences reflect typical developmental differences.

### Behavioral Findings

As often occurs, there were behavioral differences between children and adults. Children made more errors and had slower responses, and moved more in the scanner than did adults. The direct influence of performance on ERP and fMRI measures were limited in that the vast majority of trials (91% of fMRI localizer trials, 92% of fMRI and ERP letter reversal trials) involved stimuli for which no response was required. In regards to the contrast between typical and reversed letters, it seems unlikely that the worse performance of the children influenced findings because there was no interaction between age and condition (i.e., error rates and slowed responses were similarly worse in children for typical and reversed letters).

The developmental differences in brain responses also occurred in the context of specific tasks demands. Participants had to decide whether each stimulus was colored green or black, and respond only for the small minority of trials on which the letters were colored green. Thus, the orientation of each letter stimulus was independent of the required judgment, and there was no need to make explicit orientation judgments. Therefore, brain responses were unlikely to reflect higher-order cognitive processes or explicit analyses of letter orientation.

### fMRI Findings

With fMRI, adults showed extensive activation for reversed letters compared to typical letters not only in the ventral visual “word form” stream, but also in the parietal lobe, and middle and superior frontal gyri. The greater response for reversed letters may reflect greater attention being paid to relatively novel reversed letters (that are almost never seen) versus typical letters that are often seen and processed relatively fluently and automatically. In contrast, with the sole exception of a region in the right middle frontal gyrus, children exhibited activations that were greater for typical than reversed letters (the opposite of adults). Greater activation in children for typical than reversed letters occurred not only in visual areas, but also in the left superior temporal gyrus and left inferior parietal lobule, two regions thought to be engaged in phonological processing [37]–[39]. This greater activation for typical letters may reflect greater allocation of resources (i.e., less automaticity) for processing print in children as evidenced by studies reporting that children often have stronger activation than adults to words in the occipitotemporal reading network [40]–[43].

Alternatively, adults may have intentionally or incidentally rotated the reversed letters in an attempt to read them. This could explain the activation of the inferior parietal lobule and superior frontal gyrus for reversed letters compared to typical letters in adults, as these regions have been reported to be active in neuroimaging studies of mental rotation [44]–[47]. Activations may also have reflected both intentional rotations in some brain regions and incidental or automatic responses to unusual letters in other brain regions. Athough our study focused on VWFA activation related to letter processing because of that region's putative role in reading, activation differences between typical and reversed letters occurred in many brain areas in the adults.

Developmental fMRI studies comparing children and adults face a number of methodological issues. One issue is the combination of brains that differ anatomically with age into a common space for statistical analyses. For fMRI analysis, we normalized individual brains to an MNI adult template. In general, it has been shown that such normalization creates registration error that is lower than typical (including the present study) functional imaging resolution [48]. Further, the specific normalization method used in the present study (ANTs) has been shown to have registration error between children (age 4–11) and adults that is lower than our functional imaging resolution [49]. This makes it unlikely that differences between children and adults might have resulted from lower quality normalization in the children.

A second important issue in developmental fMRI is the common finding that children have more artifactual time points rejected due to motion and other sources of artifact. Indeed, we found that children had a significantly greater number of rejected fMRI time points than did adults. For several reasons, however, we believe that these age-correlated differences in outlier data points did not spuriously produce our findings. First, we carefully eliminated outlier data points, and it has been shown that such elimination can minimize age-related confounds [50], [51]. Second, both ROI and whole-brain subsidiary analyses employing artifact-matched subsets of children and adults showed the same patterns of results as the overall sample. Third, the pattern of whole-brain results (greater activation for reversed letters in adults versus greater activation for typical letters in children) is an unlikely consequence of movement. Finally, ERP measures are not sensitive to the same sources of measurement difficulty (indeed, children exhibit larger ERP responses than adults) as fMRI measures, and the ERP measures also revealed large differences between adults and children.

### ERP Findings

Children and adults first diverged in the electrophysiological pattern they showed for letters and reversed letters in the P1 component. Adults had a larger amplitude P1 response for reversed than typical letters, whereas the children had no significant difference in response to reversed and typical letters. The P1 component is thought to reflect early, low-level featural processing that is not specific to stimulus content [52]. However, some studies have found that the P1 can be modulated by meaning, with real objects engaging more attention than non-objects (e.g., [53]). In addition, P1 differences have been observed when stimuli are presented in familiar versus unfamiliar visual formats [54]. Therefore, this early difference between children and adults may reflect sensitivity in the adults to the orientation that drives more attention to the reversed letters compared to normally oriented letters, whereas in the children, this reversal is less salient.

Children and adults also exhibited significant differences in the N170 ERP component, with adults, but not children, showing a differential response to typical and reversed letters. The N170 response to words is associated with reading development. Studies have reported that pre-reading kindergartners showed no N170 differences between symbols and words in kindergarten, but by second grade typically reading children showed a left lateralized N170 difference between words and symbols; in contrast second-grade dyslexic children failed to show the N170 difference (Maurer et al., 2007; 2009). These findings support a relation between the N170 and the tuning of orthographic representations. The finding of a relation between the magnitude of the N170 response and activation in the functionally defined VWFA in the same participant is consistent with intracranial electrophysiological evidence that the N170 response is generated in the fusiform cortex [20], [55].

The few ERP studies investigating the effect of letter reversal have focused on mental rotation and later ERP components such as the N400 [17], [18]. The typical finding in these studies is a posterior negativity for mirror-reversed or rotated letters compared to normally oriented letters. The present study examined more incidental or automatic perception of reversed letters rather than intentional rotation. The general pattern of ERP findings in adults, however, is similar to a finding that adults show different P1 and N170 responses to mirror-reversed and typical letters [19].

The finding that adults showed early ERP differences for reversed versus typical letters, and that the children did not show such differences, supports the view that the developmental brain differences observed in the fMRI study are unlikely to be explained only by later-stage feed-back or rotation operations. Rather, the early ERP differences suggest that children have a less mature early-stage orthographic process in single letter identification.

## Conclusions

The present findings suggest that there is a remarkably long developmental process for the differential visual perception of typical and reversed letters. Children up to 12 years of age exhibited no P1 or N170 ERP differences for the two kinds of letters, whereas adults exhibited large and reliably greater responses for the reversed letters. FMRI revealed that the children exhibited no difference between the two kinds of letters in many brain regions, and sometimes exhibited greater activation for typical than reversed letters. In sharp contrast, the adults exhibited widespread activation for reversed relative to typical letters. These fMRI activation differences were observed in whole-brain analyses as well as ROI analyses focused on an independent functional localization of the putative VWFA, a brain region specialized for the processing of written words.

These findings are surprising because they occurred for above-average reading children after years of reading experience. These years of reading experience included countless exposures to typically oriented letters, and very few exposures to reversed letters. Yet, these children exhibited almost no difference in ERP or fMRI responses to typical versus reversed letters, whereas the adults exhibited much greater responses to the unusual reversed letters than the typical letters. The ERP findings indicate that these developmental differences are apparent within 100 msec of seeing the letters, and persist through the critical early stages of letter perception. The lack of an age correlation in the VWFA results among the children indicates that a great deal of maturation must occur through adolescence.

Reading typically involves intentional perception of individual letters in the context of whole words and surrounding text, rather than incidental perception of isolated letters. Phenomena such as the word superiority effect in which individual letters are better recognized in the context of words than as isolated letters or within nonword letter stings [56], [57] demonstrate that typical reading is a highly interactive process among lower-level letter identification processes and higher-level semantic and phonological processes. Therefore, the processing of individual letters may be less directly practiced over time than the processing of letters in the contexts of words and sentences. Perhaps the development of the perception of individual letters is functionally neglected after the earliest stages of reading acquisition as children focus on word reading. Such a focus on word-level reading cannot fully explain, however, why adult readers differentially process typical and reversed letters so quickly and in so many reading-relevant brain regions, whereas children appear to process typical and reversed letters very similarly despite years of reading experience. This extended developmental timetable is not unknown in language development. It has been reported that adult-like categorical perception of native phonemes remains immature through at least age 12 [58].

The remarkable “brain blindness” to letter orientation in children is consistent with the view that letter perception begins developmentally with visual processes that are orientation insensitive. Reading is a relatively new evolutionary skill, likely relying on object recognition abilities. Whereas object recognition is tuned to recognize objects regardless of mirror translations, this trait of the object recognition system is disadvantageous for reading. The observation that children often make reversal mistakes (not realizing letters have a correct orientation) provides ecological evidence that acquiring this skill utilizes, in some part, components of the object recognition system. Part of becoming a skilled reader involves understanding and establishing representations of letters that are orientation specific.

For object recognition, several lines of evidence have shown that the human visual system generalizes between objects and their mirror images. The ability to recognize objects from various view-points has advantages in perceiving one's environment, allowing one to identify a potential threat from many different views. In primates, single-cell recordings from the inferior temporal (IT) cortex show invariance to mirror images [59]. Brain imaging studies further support that the visual system generalizes across mirror reversals for objects [60], [61].

While mirror generalization is an adaptation for viewing naturalistic surroundings, it is a handicap when it comes to reading. In the majority of writing systems, orientation matters for letter identity. Therefore, learning to read requires selectively unlearning mirror generalization for letters, the most basic level orthographic representations involved in reading. The present study indicates that “unlearning” visual mechanisms that are orientation-insensitive and fruitful for object recognition in general requires a surprisingly long developmental period that extends to at least early adolescence. Future studies could include symbolic stimuli other than letters to gain a better idea about underlying mechanisms involved in orientation sensitivity in beginning readers.
